# Gateways not gatekeepers – reaching seldom-heard groups to gather public health community insights: Mabel Lie

**DOI:** 10.1093/eurpub/ckac130.030

**Published:** 2022-10-25

**Authors:** M Lie, S Visram, M Cheetham, A Christie, P Hodgson, J Jasperse, M Logan

**Affiliations:** 1 Population Health Sciences Institute, Newcastle University, Newcastle upon Tyne, UK; 2 Department of Nursing, Midwifery and Health, Northumbria University, Newcastle upon Tyne, UK; 3 Public Health, South Tyneside Council, South Shields, UK

## Abstract

**Background:**

Each local authority in England must develop a Health and Wellbeing Strategy (HWS) in collaboration with NHS partners to plan and support delivery of local improvements in health and wellbeing. HWSs often draw on diverse sources but few are informed by consultative exercises involving citizens. South Tyneside Council in Northern England sought to ensure their new HWS was community-informed, specifically including seldom-heard groups and individuals. Specific objectives of this community insights research were to:

1.Target sampling and recruitment activities at typically marginalized, vulnerable or otherwise underrepresented groups

2.Explore the health and wellbeing-related views and priorities of these groups to address health inequalities

**Methods:**

A mapping exercise was undertaken to identify organisations who might act as gatekeepers to accessing participants from underrepresented groups. Focus groups were held in settings-based venues where members would be comfortable and known to one another. Representatives of voluntary and community sector (VCS) organisations often helped to co-facilitate the discussions.

**Results:**

119 participants took part in 16 group discussions. Three were held online, two were outdoors, while 11 involved community venues where the groups regularly met. We reached older and younger people, minority ethnic groups, and vulnerable men and women, including residents who had experienced homelessness, mental health issues, substance misuse, offending, domestic violence and learning disabilities. Participants were largely concerned with the wider determinants of health (such as poverty, employment, and leisure spaces), shifting the narrative away from individual lifestyle factors that tend to be the focus of much public health discourse.

**Conclusions:**

Gatekeepers from the VCS were essentially gateways, enabling us to include underrepresented voices in local consultation processes and generate new insights to inform the South Tyneside HWS.

**Key messages:**

- Public health strategy development can address health inequalities through community-informed consultative exercises involving seldom heard members of vulnerable communities.
- Community consultations seeking to reach typically underrepresented groups require gatekeepers acting as gateways, and meeting participants where they are comfortably located at community venues.

